# Efficacy of a hydrogen–oxygen generator in treating cigarette smoke–induced chronic obstructive pulmonary disease in rats

**DOI:** 10.1016/j.crtox.2024.100214

**Published:** 2024-12-28

**Authors:** Wan-Ting Huang, Tzong-Jih Cheng, Lin-Hsiang Huang, Yung-Te Hou

**Affiliations:** a Department of Biomechatronics Engineering, National Taiwan University, No. 1, Sec. 4, Roosevelt Road, Taipei 10617, Taiwan; b Animal Resource Center, National Taiwan University, No. 118, Ln. 155, Sec. 3, Keelung Road, Taipei 10673, Taiwan; c North-vision Tech. Inc. No. 15, Gongye E. 2nd Rd., East Dist., Hsinchu 300, Taiwan

**Keywords:** Hydrogen–oxygen generator, Steroid, Chronic obstructive pulmonary disease, Bronchoalveolar lavage fluid, Cigarette smoke, Rat model

## Abstract

Current treatments for chronic obstructive pulmonary disease (COPD), a common respiratory condition, include oxygen therapy and steroids for temporary relief. In this study, we established a rat model of cigarette smoke (CS)–induced COPD and investigated the benefits of a hydrogen–oxygen generator in this model. CS–exposed rats were treated using either a hydrogen–oxygen generator or a steroid. A hydrogen–oxygen generator reduced the neutrophil, lymphocyte, and eosinophil counts compared to natural recovery, whereas steroid treatment increased the total white blood cell, neutrophil, lymphocyte, monocyte and eosinophil counts. Furthermore, the mean linear intercept and the mean alveolar number were 59.8%, and 188.3%, respectively, after treatment with the generator, compared to the values observed with natural recovery. Finally, the generator increased the tricuspid annular plane systolic excursion values by 113.1% compared with the values in natural recovery. Our findings indicate successful establishment of a rat model of CS–induced COPD and demonstrate the potential benefits of using a hydrogen–oxygen generator for COPD patients.

## Highlights

• Our findings indicate successful establishment of a rat model of CS–induced COPD.
• The hydrogen–oxygen generator outperformed steroid treatment in conferring the recovery benefit for CS–induced COPD rats.
• TAPSE helps detect potential damage to the right ventricular function in patients with COPD.

## Graphical abstract

**Figure:**
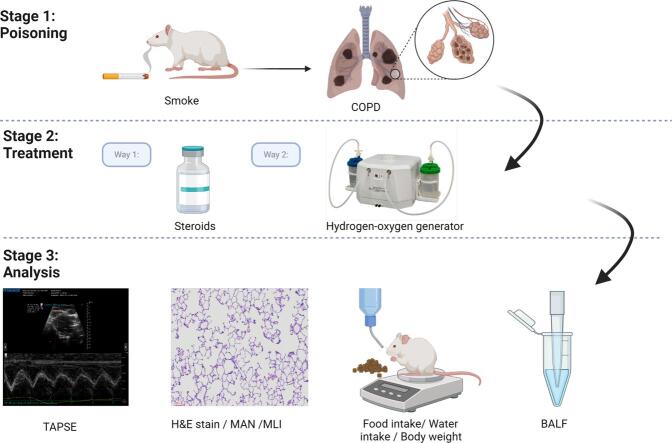

## 1 Introduction

Chronic obstructive pulmonary disease (COPD), a widespread chronic inflammatory condition, is a preventable and treatable but irreversible respiratory illness (Alqahtani, 2022). Key features of COPD include tracheal inflammation, obstructive asthma, and emphysema, which ultimately lead to respiratory distress and even death (Angelis et al., 2014). In 2023, the Global Initiative for Chronic Obstructive Lung Disease proposed various treatment modalities, such as bronchodilators, inhaled corticosteroids, inhibitors targeting inflammatory mediators, oxygen therapy, and other pulmonary protective strategies (Agustí et al., 2023, Hanania et al., 2005). However, these approaches only offer symptomatic relief and cannot reverse lung damage (Hansen et al., 1999, Teramoto, 2007). Therefore, identifying novel approaches to ameliorating COPD symptoms is imperative (Cazzola et al., 2007).

Hydrogen, an inert gas with low water solubility, is present in various organs, including the liver, kidneys, and lungs. This gas exerts antioxidative and anti-inflammatory effects (Ichihara et al., 2015). These effects are attributed to the hydrogen’s small molecular weight, which allows it to penetrate biological membranes and access cell cytoplasm and organelles; notably, hydrogen can penetrate even those areas that are typically less reachable by most antioxidants—for example, the blood–brain barrier (Ohta, 2012). Furthermore, hydrogen exhibits higher levels of compatibility with various tissues than do other oxidants (Ohta, 2011, Tian et al., 2021). When inhaled, hydrogen diffuses directly from the alveoli into the plasma and exerts systemic effects through blood circulation, without affecting physiological systems and parameters, such as the nervous system, pulmonary function, and electrocardiogram (Cole et al., 2021). Given that hydrogen can effectively regulate mitochondrial oxidative stress, it is considered to be an ideal reductant in cases where the electron transport chain is impaired due to cellular damage (Annesley & Fisher, 2019).

Hydrogen is typically used in lung therapy to inhibit the generation of free radicals, thereby mitigating lung inflammation, rather than directly restoring damaged lung tissue (Chen et al., 2018). Kohama et al. highlighted hydrogen’s ability to alleviate oxidative damage in the lungs (Kohama et al., 2015). Liu et al. demonstrated the efficacy of hydrogen in the treatment of COPD; this gas readily reaches the lungs and then exerts therapeutic effects (Liu et al., 2011). However, the use of high hydrogen concentrations is associated with elevated risks of explosion and cellular toxicity (Rigas & Sklavounos, 2008). Oxygen therapy, a common strategy for COPD treatment, increases the partial pressure of oxygen in the blood, thereby mitigating hypoxemia (Brill & Wedzicha, 2014). However, high rates of oxygen flow may lead to the retention of carbon dioxide and pose a risk of explosion (Tarpy & Celli, 1995). Combining hydrogen and oxygen may help regulate their concentrations and thus harness their individual therapeutic effects (Russell et al., 2024). Zhou et al. used a mixture of hydrogen and oxygen to treat patients with tracheal stenosis; although this mixture did not fully expand the trachea, it alleviated patient discomfort (Zhou et al., 2018). Similarly, Zheng et al. used a mixture of hydrogen and oxygen to effectively treat acute exacerbation of COPD; the mixture outperformed oxygen therapy in terms of efficacy (Zheng et al., 2021). Thus, hydrogen–oxygen mixtures appear to hold promise in the treatment of pulmonary diseases.

The use of hydrogen–oxygen mixtures gained prominence during the COVID-19 pandemic. A human trial conducted during this period demonstrated the efficacy of these mixtures (Guan et al., 2020). Patients with COVID-19-induced acute pneumonia who inhaled a mixture of hydrogen and oxygen (from a medical hydrogen–oxygen generator nebulizer) exhibited improved clinical outcomes (Zheng et al., 2021). However, very few animal studies have explored the mechanisms underlying the therapeutic benefits of hydrogen–oxygen mixtures against COPD. Therefore, this study was conducted to establish a rat model of cigarette smoke (CS)–induced COPD and elucidate the precise pathogenic mechanisms of COPD. We further compared a hydrogen–oxygen generator with steroid treatment in terms of their effects on bronchoalveolar lavage fluid (BALF), histological parameters, and tricuspid annular plane systolic excursion (TAPSE) values.

## 2 Methods

### 2.1 Custom-made CS exposure device

#### 2.1.1 Gas sensor selection

Since tobacco strength (amounts of tar, nicotine, and carbon monoxide) and various additives influence the concentration of PM (PM_10_, PM_2.5_, PM_1_) (Braun et al., 2019). PM_2.5_ sensors (HM3301; Taiwan Intelligent Sensor Technology, Taiwan) and carbon monoxide sensors (MQ-7; Taiwan Intelligent Sensor Technology) were used for detecting gas components in a custom-made CS exposure device. The sensors utilized a laser-based digital universal particulate matter concentration sensor capable of detecting the number of suspended particles ranging from 0.3 to 10 μm in a given unit volume of air, thus determining the particulate matter concentration. This sensor is equipped with an internal microcontroller unit (MCU), which automatically calculates and outputs data through a digital interface. Additionally, it can provide mass data for each particle type. The calibration method employed a laser particle counter (A2400) as the standard for testing, using the least squares method to construct a regression equation for sensor calibration and data conversion. The entire automated control process was implemented using Arduino IDE (version 1.8.19). Component drawings were generated using Solidworks (version 2017). Images were analyzed using ImageJ.

### 2.2 CS–induced COPD rat model

Several studies have used rats as modes for cigarette smoke-induced COPD (Wang et al., 2018, Wang et al., 2014). Additionally, secondhand smoke, also known as passive smoking, includes the inhalation of smoke emitted from burning tobacco products and the exhaled gases from smokers. Passive exposure to CS is associated with a higher incidence of asthma and chronic bronchitis (Radon et al., 2002). Given the high prevalence of CS exposure, particularly in outdoor public areas (Yousuf et al., 2020), we used Sprague Dawley rats with passive CS exposure to develop a COPD model.

Male Sprague Dawley (SD) rats, initially 6 weeks old, were randomly divided into four groups: control group (nonsmoking), smoking group, smoking group treated using the hydrogen–oxygen generator, and smoking group treated with a steroid. The rats in the smoking groups were exposed to passive smoke in a fumigation chamber (Length × width × height = 27 × 22 × 20 cm) once a day for 3 months. The control group comprised unexposed rats housed in under regular air conditions, whereas the smoking groups involved burning six cigarettes for 30 min (5 min per cigarette), simulating human smoking behavior. Cigarettes were ignited using a lighter and placed in the cigarette burning area (Length × width × height = 8 × 22 × 20 cm) to burn naturally. The smoke from the cigarette burning area was directed into the fumigation chamber by using a motor, and the concentration of CS was regulated by adjusting the chamber valves and motor voltage (decreasing the voltage reduced the motor speed and air extraction). Ventilation and exhaust systems were established in the laboratory to ensure adequate air circulation and avoid indoor CS accumulation. The CS exposure device was constructed using an acrylic box equipped with ventilation holes to simulate open-air environments. To further avoid hypoxia during CS exposure, oxygen was supplied into the chamber by using an air pump. Sensors were installed on this device to measure the concentrations of harmful gases—PM_2.5_ and carbon monoxide. After 3 months of CS exposure, the rats received relevant treatment for 3 months as described in the following section.

### 2.3 Hydrogen–oxygen generator for CS–induced COPD rat model

Some rats were treated using a hydrogen–oxygen generator (Lifecare-1000, North-vision Tech. Inc., Taiwan), which was equipped with a proton exchange membrane for electrolyzing ultrapure water to produce 1000 cc/min of hydrogen and 500 cc/min of oxygen. The device has been certified by the Taiwan Food and Drug Administration as a class II medical device for its oxygen portion, with hydrogen being an additional option. The rats were then placed in the treatment chamber (Length × width × height = 50 × 50 × 50 cm), which was connected with a pipeline from the hydrogen–oxygen generator. The generator continuously supplied a mixture of hydrogen and oxygen into the treatment chamber for 2 h (this exposure was repeated every day for 3 months). During the 2-h period, the air in the treatment chamber comprised approximately 66 % hydrogen and 33 % oxygen.

**Scheme 1:**
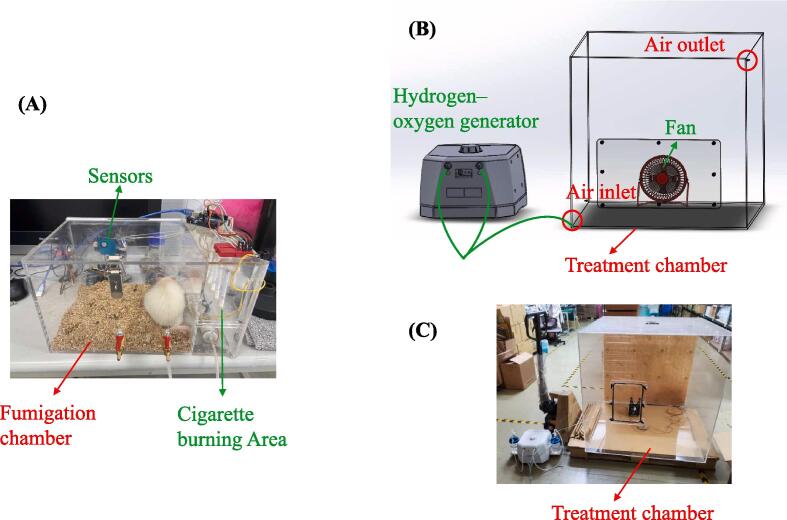
Custom-made CS exposure device. (A) Rats were exposed to CS in the fumigation chamber (on the left) featuring internal sensors. The right side housed the cigarette burning area; (B) The treatment chamber was equipped with a small fan to enhance convection and prevent gas layering within the chamber, considering the lightness of hydrogen gas. CS, cigarette smoke. (C) The actual photos of the treatment chamber, which was connected with a pipeline from the hydrogen–oxygen generator. Fumigation chamber: Length × width × height = 27 × 22 × 20 cm. Cigarette burning area: Length × width × height = 8 × 22 × 20 cm. Treatment chamber: Length × width × height = 50 × 50 × 50 cm.

The second treatment involved steroid therapy at a concentration of 1 mg/kg/day (similar to the concentration in Horváth et al., 1998). In this study, triamcinolone acetonide (TAI YU Chemical & Pharmaceutical Co., Ltd; Taiwan) was used as the steroid. A custom-made nebulizer was employed, featuring a 16 mm ultrasonic nebulization plate with a micropore area of 4.5 mm and 600 micropores. The nebulizer operates by using high-frequency electronic oscillations to break down liquid water molecules, producing a natural mist. Triamcinolone acetonide was dissolved in PBS, and this solution was placed into the custom-made nebulizer. The rats were then exposed to the nebulized solution in the treatment chamber for 2 h each day, continuously for 3 months. Scheme 1 illustrates the structure of our custom-made CS exposure device.

The rats used in this study were sourced from the National Laboratory Animal Center (Taiwan). The experimental rats were housed in clean cages (26 × 45 × 26 cm) and closely monitored at a constant temperature of 25 °C. They were maintained on a 12-h light/dark cycle with access to an adequate supply of food and water. The food provided to the experimental rats was MFG (Oriental Yeast Co., Ltd, Japan). The experimental protocol was reviewed and approved by the Ethics Committee on Animal Experiments at National Taiwan University (approval number: NTU-110-EL-00101).

### 2.4 Human equivalent dose

Following the FDA guidance for industry on body surface area conversion (Guidance for Industry Estimating the Maximum Safe Starting Dose in Initial Clinical Trials for Therapeutics in Adult Healthy Volunteers, 2005). In our recent study, assuming a rat's body weight is 480 g and a human's body weight is 60 kg, and the administered dose for the rat is 1 mg/kg, the rat dose can be converted to the human equivalent dose (HED) using the following formula based on body surface area: The method for calculating the values of human km and animal km was based on the previous study (Nair & Jacob, 2016).

$$HED\left(mg/kg\right)=AnimalDose\left(mg/kg\right)\times \frac{\mathrm{HumanKm}}{\mathrm{AnimalKm}}$$

(1) $$HED\left(mg/kg\right)=1\left(mg/kg\right)\times 0.2\approx 0.2mg/kg$$

Next, we convert the HED to the actual human dose. Assuming a human body weight of 60 kg, the human dose calculation is as follows:

$$HumanDose\left(mg\right)=HED\left(mg/kg\right)\times HumanBodyWeight\left(kg\right)$$

(2) $$HumanDose\left(mg\right)=0.2\left(mg/kg\right)\times 60\left(kg\right)\approx 12mg$$

### 2.5 BALF analysis

A BALF analysis was previously conducted in a rat model of CS–induced COPD to quantify neutrophils, macrophages, and lymphocytes (Zheng et al., 2009). These procedures were performed by the Taiwan Mouse Clinic (TMC) and followed previously established protocols (Song et al., 2010). In brief, the rats were euthanized with isoflurane. With the help of scissors, a skin incision was made from the abdomen to the neck. Then, the skin was gently pulled apart with forceps, exposing the chest and neck The muscles near the neck were carefully separated to unveil the trachea. A nylon string was placed under the trachea, and the ribs were cautiously cut to expose the heart and lungs (avoiding any cuts to the trachea or lungs). A 19G indwelling needle was inserted into the trachea for BALF extraction; the needle was tied to the trachea with the nylon string. Subsequently, PBS (30 mL/kg) was slowly injected into the lungs and then aspirated out; this procedure was repeated three times. The collected BALF was placed in a 15-mL conical tube and centrifuged at 300 × *g* for 5 min at 4℃. The supernatant was removed, and 1 mL of PBS was added to the pellet. The cells were stained using Turk’s solution and counted on a hemocytometer, and the protein contents of the BALF and serum were determined using a bicinchoninic acid protein assay kit (Pierce, Rockford, IL, USA).

### 2.6 Hematoxylin–eosin staining

Hematoxylin–eosin staining was previously performed to comprehensively examine lung histology in a rat model of CS-induced COPD (Zheng et al., 2009). To achieve deep anesthesia, excess isoflurane was administered to the rats, covering their mouths and noses for approximately 20 s; then, the rats were examined for a response to toe-pinching. Once fully anesthetized, the animals were placed in the supine position for the further euthanasia. A midline incision was made from below the diaphragm, extending along the ribs to the right, exposing the heart. A small incision was made in the left ventricle, and a needle was inserted into the aorta and clamped while incising the right atrium to enable flow. Subsequently, the heart was perfused with 15 mL of 4 % paraformaldehyde (fixative solution) for 5 min. Any limb tremors observed during perfusion were monitored, given that they could indicate neurogenic or muscular cross-linking–induced tremors. After perfusion, tissues were dissected and fixed in 4 % paraformaldehyde or a 10 % formalin solution at a ratio of 1:20 and incubated under shaking conditions for 24 h to prevent protein denaturation and cellular breakdown due to bacterial or endogenous apoptosis, thereby maintaining the original tissue structure.

### 2.7 TAPSE

TAPSE is a commonly used clinical metric for assessing the right ventricle systolic function. In M−mode echocardiography, TAPSE values are estimated by aligning the lateral tricuspid annulus with the ventricular apex in the apical four-chamber view and measuring the lateral annular displacement (O'Riordan et al., 2023). The following parameters were used in TAPSE measurement: the Prospect T1 machine (S-Sharp Corporation, New Taipei City, Taiwan), PB207e probe, and 13–20 MHz frequency. The experimental protocols and analytical methods were adapted from another study (Rosas et al., 2023).

### 2.8 Statistical analysis

In all quantitative experimental results, the data are presented as mean ± standard deviation. For significance comparisons between two experimental groups, an unpaired two-tailed Student's *t*-test was used, and Microsoft Excel 2017 was employed for the analysis. For significance comparisons among multiple experimental groups, one-way analysis of variance (one-way ANOVA) was performed, followed by Fisher's post-hoc test to further clarify the significance between groups, using OriginPro 2024b for analysis. A p-value of less than 0.05 was considered statistically significant.

## 3 Results

### 3.1 Body condition

**Fig. 1:**
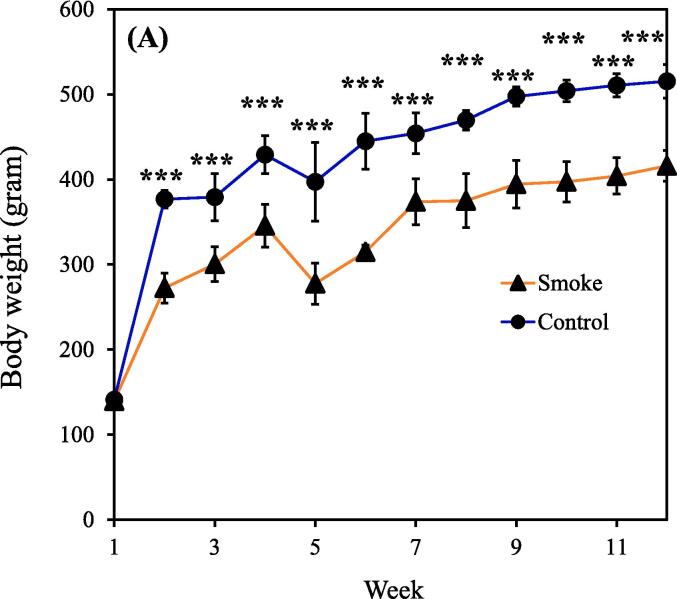

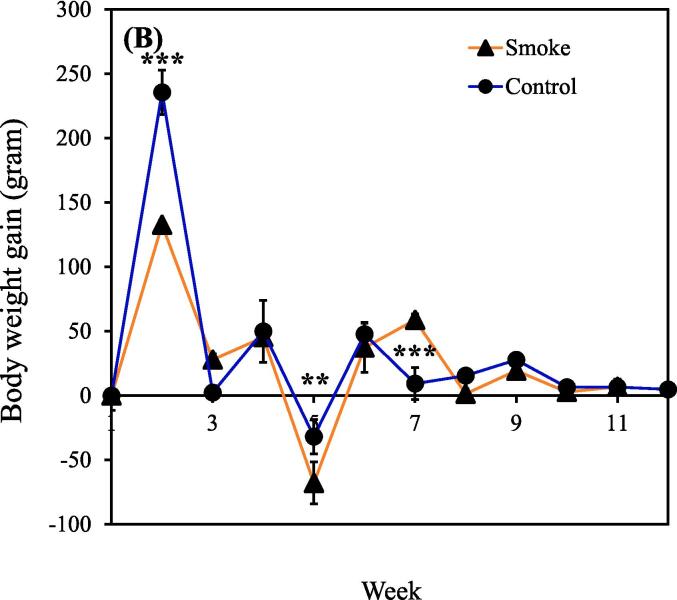

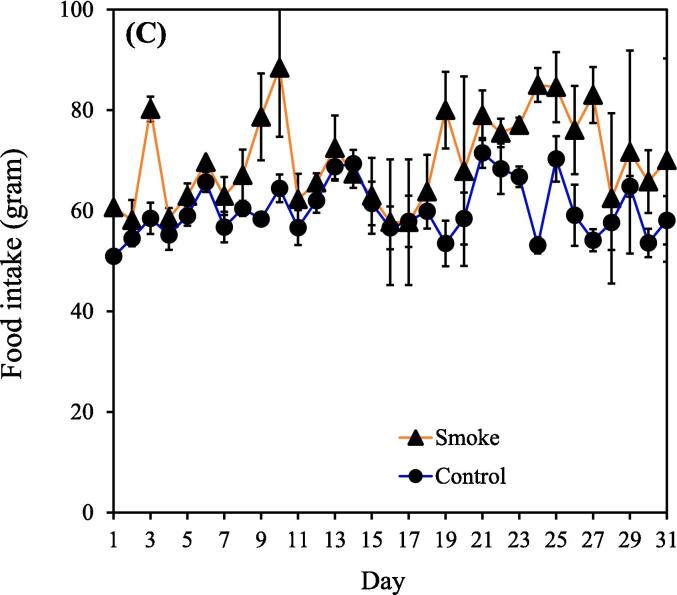

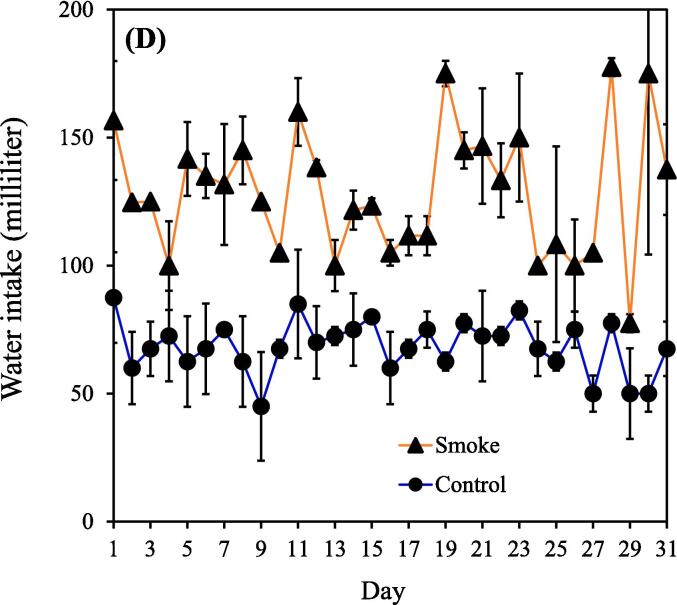
(A) Body weight in the smoking and nonsmoking groups. (B) Body weight gain in the smoking and nonsmoking groups. (C) Food intake in the smoking and nonsmoking groups. (D) Water intake in the smoking and nonsmoking groups. CS, cigarette smoke. (**p < 0.01, ***p < 0.001 compared with the smoking group).

The concentrations of PM_2.5_ and carbon monoxide generally remained consistent throughout CS exposure, indicating the ability of our device to consistently release toxic substances (Supplemental Fig. S1A-B). It should be noted that the findings presented in this study are based on a single experimental trial. Notably, significant differences in body weight between the smoking (n = 9) and control (n = 5) groups became apparent week 2 onward. The body weight estimates of the smoking group from weeks 1 to 12 were 139.7 ± 6.1, 272.4 ± 17.6, 300.3 ± 20.4, 345.6 ± 25.0, 277.7 ± 24.1, 314.9 ± 7.8, 373.7 ± 27.0, 375.1 ± 31.7, 394.4 ± 28.0, 397.2 ± 23.8, 404.0 ± 21.4, and 416.0 ± 18.1 g. During this period, the body weight estimates of the control group (nonsmoking group) were 141.2 ± 3.5, 376.8 ± 10.4, 379.2 ± 27.8, 429.1 ± 22.3, 397.2 ± 46.4, 444.9 ± 33.0, 454.2 ± 23.8, 469.7 ± 11.4, 497.5 ± 11.4, 504.0 ± 12.6, 510.6 ± 13.5, and 515.4 ± 19.6 g (Fig. 1A). Fig. 1B demonstrates that body weight gain throughout the study was consistent between groups, with most of the difference in body weight occurring during the first two weeks of CS exposure. Additionally, the food intake and water intake of rats in the CS group were higher than those in the control group within the first month (Fig. 1C-D). This observation appears contradictory to the results shown in Fig. 1A.

### 3.2 Pretreatment BALF

The control group consisted of 3 rats (n = 3), while the CS exposure groups were divided based on exposure duration: 1 month (n = 3), 2 months (n = 2), and 3 months (n = 2). Exposure to CS treatment resulted in an increased total count of white blood cells, neutrophils, lymphocytes, and monocytes in the BALF compared to the control group. Conversely, the count of eosinophils in the BALF was slightly decreased relative to the control group after CS exposure for 3 months.

**Fig. 2:**
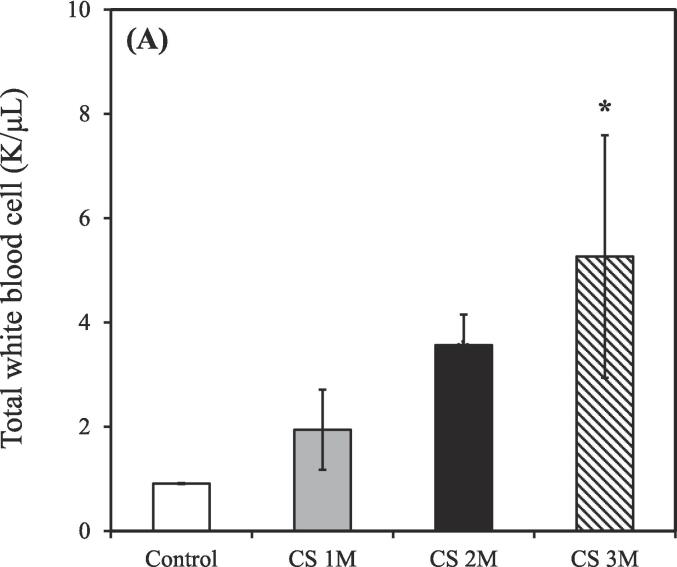

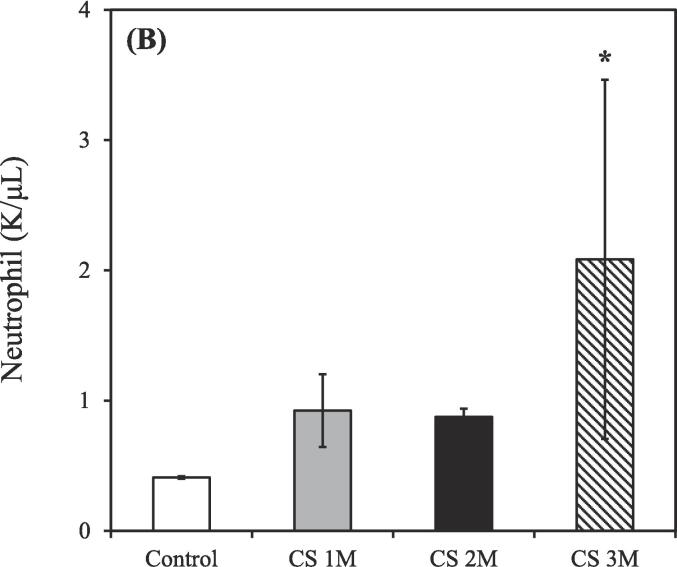

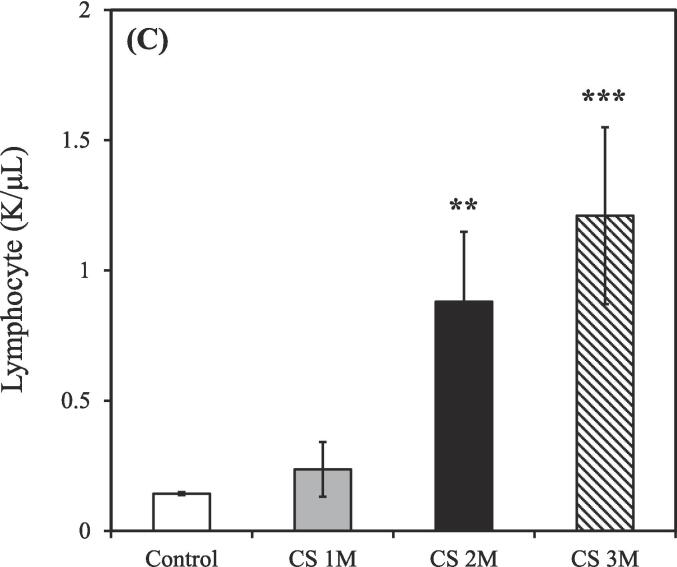

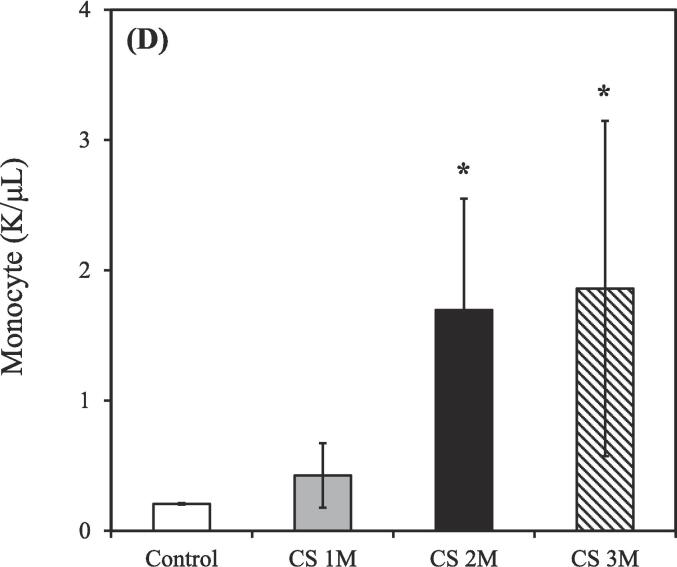

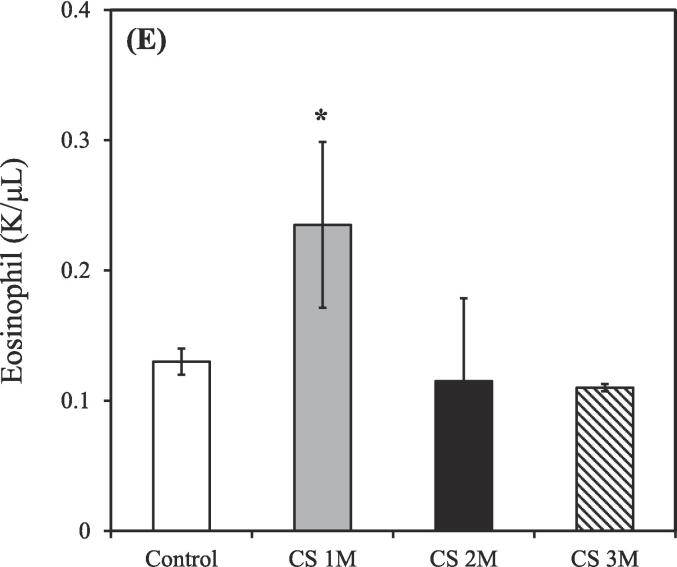
Differential counts of white blood cells in the bronchoalveolar lavage fluid of CS–exposed rats at various time points. (A) Total white blood cells; (B) Neutrophils; (C) Lymphocytes; (D) Monocytes; (E) Eosinophils. CS exposure durations: 1, 2, and 3 months. CS, cigarette smoke. [*p < 0.05, **p < 0.01, ***p < 0.001 compared with the nonsmoking (control) group].

As shown in Fig. 2A, the total count of white blood cells in the BALF of the control group was 0.91 ± 0.01 K/μL. However, after CS exposure for 1, 2, and 3 months, the total white blood cell count increased to 1.94 ± 0.77, 3.57 ± 0.59, and 5.27 ± 2.32 K/μL, respectively.

As shown in Fig. 2B, the count of neutrophils in the BALF of the control group was 0.41 ± 0.01 K/μL. However, following after CS exposure for 1, 2, and 3 months, the neutrophil count increased to 0.92 ± 0.28, 0.88 ± 0.06, and 2.10 ± 1.36 K/μL, respectively.

As shown in Fig. 2C, the count of lymphocytes in the BALF of the control group was 0.14 ± 0.01 K/μL. However, after CS exposure for 1, 2, and 3 months, the lymphocyte count increased to 0.24 ± 0.11, 0.88 ± 0.27, and 1.21 ± 0.34 K/μL, respectively.

As shown in Fig. 2D, the count of monocytes in the BALF of the control group was 0.21 ± 0.01 K/μL. However, after CS exposure for 1, 2, and 3 months, the monocyte count increased to 0.42 ± 0.18, 1.70 ± 0.86, and 1.86 ± 1.29 K/μL, respectively.

As shown in Fig. 2E, the count of eosinophils in the BALF of the control group was 0.13 ± 0.01 K/μL. However, after CS exposure for 1, 2, and 3 months, the eosinophil count decreased to 0.24 ± 0.05, 0.12 ± 0.06, and 0.11 ± 0.00 K/μL, respectively.

### 3.3 Posttreatment BALF

After the 3-month CS exposure, the rats were treated using either a hydrogen–oxygen generator or a steroid for an additional 3 months. BALF samples were reanalyzed after the treatment. The control group (0 M and 6 M) consisted of 3 rats (n = 3), while the CS exposure groups were divided based on different treatment: CS (n = 2), CS + natural recovery (n = 3), CS + hydrogen–oxygen (n = 2), and CS + steroid (n = 3). The smoking group treated using the hydrogen–oxygen generator [CS (3 M) + hydrogen–oxygen (3 M)] showed a decreased total count of white blood cells, neutrophils, lymphocytes, and eosinophils in the BALF compared to the smoking group treated with a steroid [CS (3 M) + steroid (3 M)]. Additionally, this group also exhibited a decreased count of lymphocytes and eosinophils in the BALF compared to the untreated smoking group [CS (3 M) + natural recovery (3 M)].

**Fig. 3:**
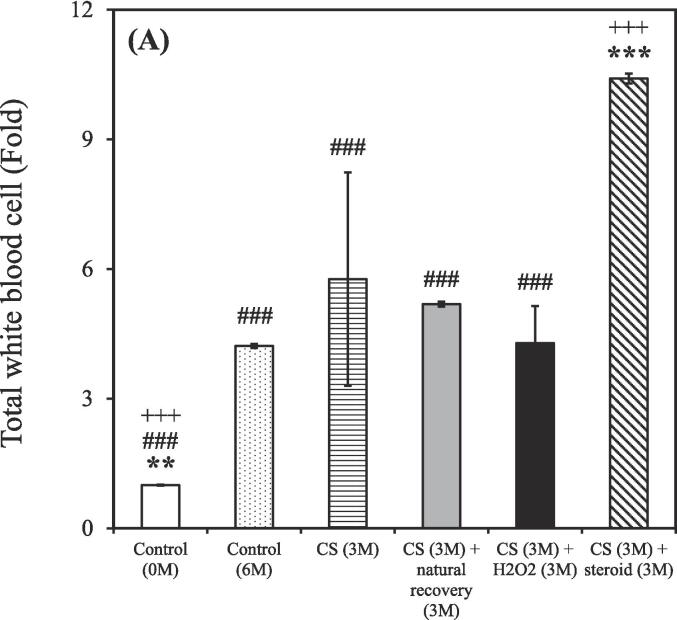

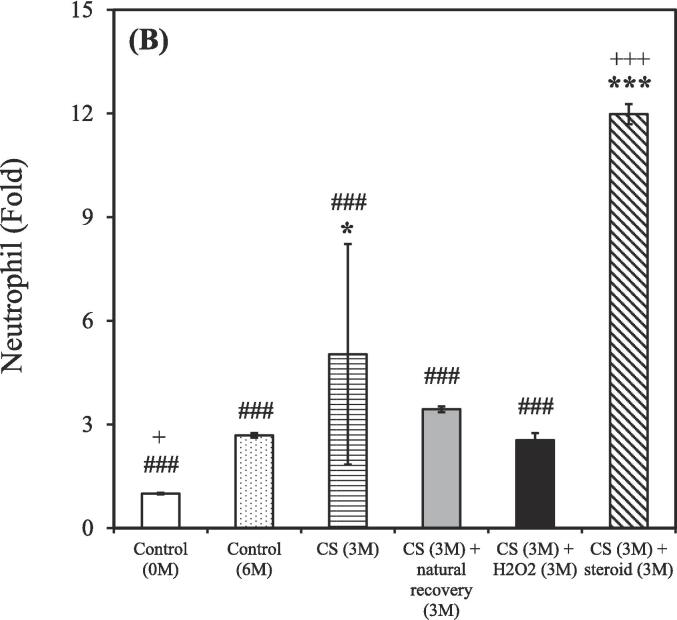

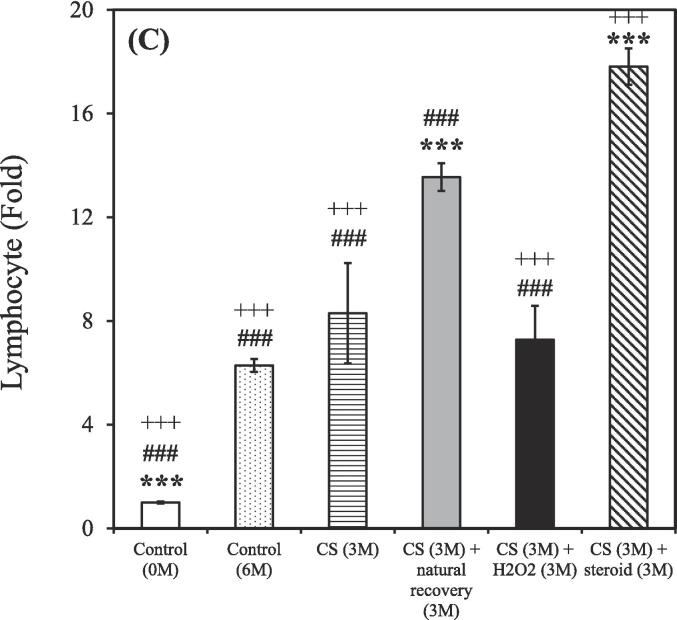

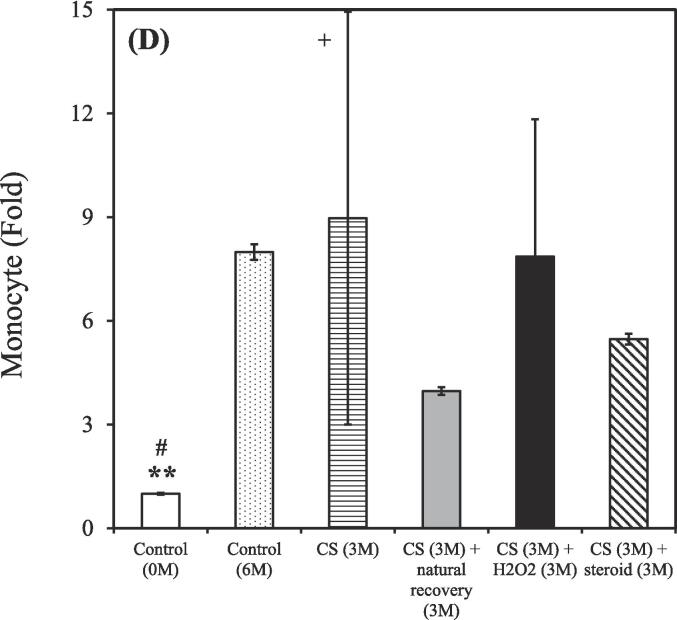

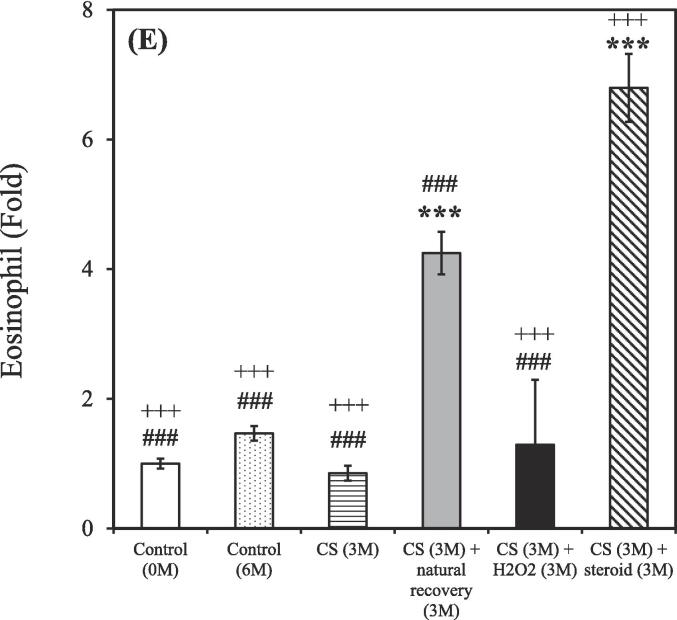

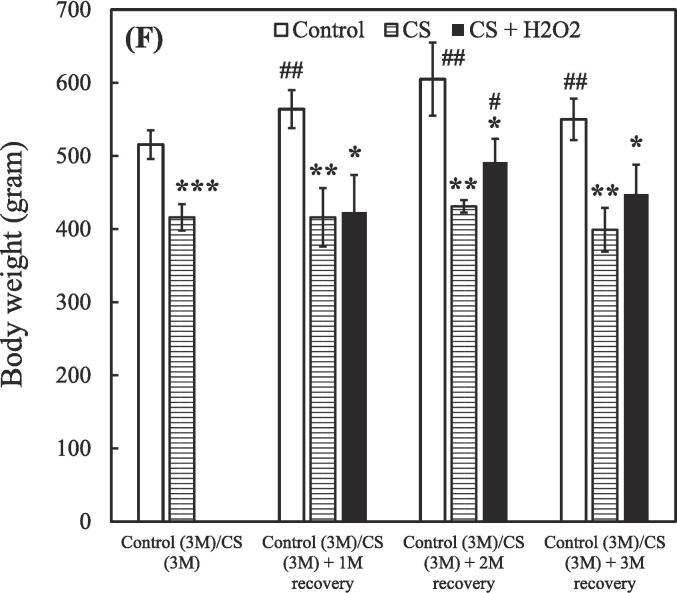
After 3-month CS exposure, the rats were subjected to different treatments and the white blood cell in the bronchoalveolar lavage fluid were counted at various time points. (A) Total white blood cells; (B) Neutrophils; (C) Lymphocytes; (D) Monocytes; (E) Eosinophils. Treatment duration: 3 months. CS, cigarette smoke. The data obtained from the analysis were quantified, normalized to the data of the untreated nonsmoking group [Control (0 M)], and then calculated as the fold change. (*p < 0.05, **p < 0.01, ***p < 0.001 compared with the smoking group treated using the hydrogen–oxygen generator [CS (3 M) + hydrogen–oxygen (3 M)]; *^#^p* < 0.05, *^###^p* < 0.001 compared with the smoking group treated with a steroid [CS (3 M) + steroid (3 M)]; *^+^p* < 0.05, *^+++^p* < 0.001 compared with the untreated smoking group [CS (3 M) + natural recovery (3 M)]). (F) Body weight in the smoking and nonsmoking groups after treatment with a hydrogen–oxygen generator. (*p < 0.05, **p < 0.01, ***p < 0.001 compared with the control group; *^#^p* < 0.05, *^##^p* < 0.01 compared with the smoking group).

A shown in Fig. 3A, the total counts of white blood cells in the BALF were 1.00 ± 0.01, 4.22 ± 0.05, 5.77 ± 2.47, 5.19 ± 0.06, 4.28 ± 0.86, and 10.41 ± 0.11 fold in the untreated nonsmoking group [Control (0 M)], untreated nonsmoking group [Control (6 M)], smoking group [CS (3 M)], untreated smoking group [CS (3 M) + natural recovery (3 M)], smoking group treated using the hydrogen–oxygen generator [CS (3 M) + hydrogen–oxygen (3 M)], and smoking group treated with a steroid [CS (3 M) + steroid (3 M)], respectively.

As shown in Fig. 3B, the counts of neutrophils in the BALF were 1.00 ± 0.02, 2.68 ± 0.07, 5.03 ± 3.19, 3.44 ± 0.08, 2.55 ± 0.21, and 11.98 ± 0.29 fold in the untreated nonsmoking group [Control (0 M)], untreated nonsmoking group [Control (6 M)], smoking group [CS (3 M)], untreated smoking group [CS (3 M) + natural recovery (3 M)], smoking group treated using the hydrogen–oxygen generator [CS (3 M) + hydrogen–oxygen (3 M)], and smoking group treated with a steroid [CS (3 M) + steroid (3 M)], respectively.

As shown in Fig. 3C, the counts of lymphocyte in the BALF were 1.00 ± 0.04, 6.29 ± 0.25, 8.30 ± 1.94, 13.55 ± 0.53, 7.28 ± 1.30, and 17.81 ± 0.70 fold in the untreated nonsmoking group [Control (0 M)], untreated nonsmoking group [Control (6 M)], smoking group [CS (3 M)], untreated smoking group [CS (3 M) + natural recovery (3 M)], smoking group treated using the hydrogen–oxygen generator [CS (3 M) + hydrogen–oxygen (3 M)], and smoking group treated with a steroid [CS (3 M) + steroid (3 M)], respectively.

As shown in Fig. 3D, the counts of monocyte in the BALF were 1.00 ± 0.03, 7.99 ± 0.23, 8.97 ± 5.97, 3.97 ± 0.11, 7.86 ± 3.97, and 5.47 ± 0.16 fold in the untreated nonsmoking group [Control (0 M)], untreated nonsmoking group [Control (6 M)], smoking group [CS (3 M)], untreated smoking group [CS (3 M) + natural recovery (3 M)], smoking group treated using the hydrogen–oxygen generator [CS (3 M) + hydrogen–oxygen (3 M)], and smoking group treated with a steroid [CS (3 M) + steroid (3 M)], respectively.

As depicted in Fig. 3E, the counts of eosinophil in the BALF were 1.00 ± 0.08, 1.47 ± 0.11, 0.85 ± 0.11, 4.25 ± 0.33, 1.29 ± 1.00, and 6.80 ± 0.52 fold in the untreated nonsmoking group [Control (0 M)], untreated nonsmoking group [Control (6 M)], smoking group [CS (3 M)], untreated smoking group [CS (3 M) + natural recovery (3 M)], smoking group treated using the hydrogen–oxygen generator [CS (3 M) + hydrogen–oxygen (3 M)], and smoking group treated with a steroid [CS (3 M) + steroid (3 M)], respectively.

Fig. 3F shows how treatment with a hydrogen–oxygen generator affects body weight gain after the cessation of CS inhalation. The body weight of the control group was 515.4 ± 19.6 g at 3 months, 564.0 ± 26.0 g at 4 months, 605.3 ± 49.3. g at 5 months, and 550.5 ± 31.8 g at 6 months, respectively. Additionally, the body weight of the CS (3 M) group was 416.0 ± 18.1 g. However, following natural recovery for 1, 2, and 3 months, the body weight changed to 416.0 ± 38.6 g, 431.0 ± 8.5 g, and 399.0 ± 29.5 g, respectively. In contrast, following treatment with the hydrogen–oxygen generator for 1, 2, and 3 months, the body weight changed to 423.0 ± 50.9 g, 493.5 ± 29.0 g, and 448.0 ± 39.6 g, respectively. These results indicate the therapeutic effect of using a hydrogen–oxygen generator for COPD. Previous studies have also mentioned that the recovery of body weight indicates a positive therapeutic effect on the rat’s general well-being (Wang et al., 2018), which is consistent with our findings.

### 3.4 Histopathology

The histopathological findings highlight the efficacy of the hydrogen–oxygen generator in treating COPD and mitigating its detrimental effects on lung tissue.

**Fig. 4:**
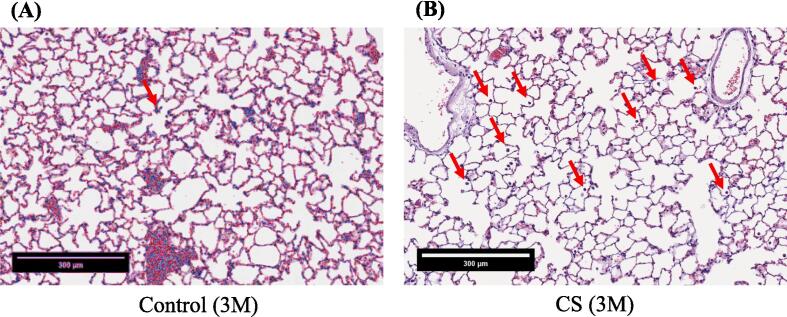

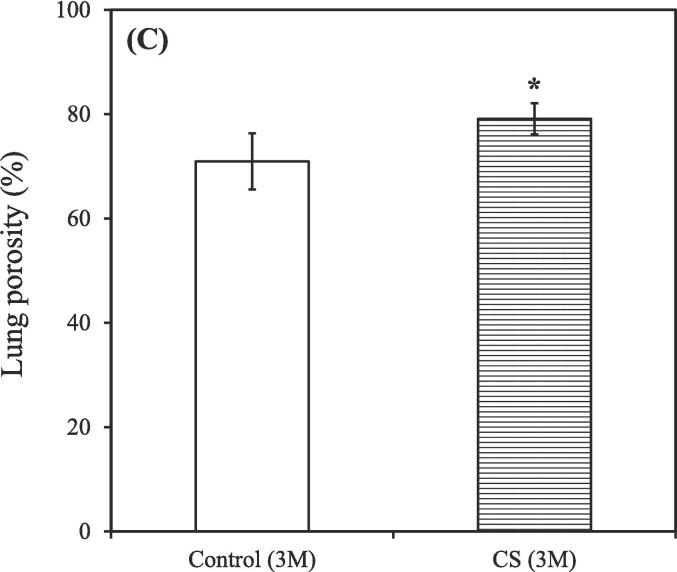

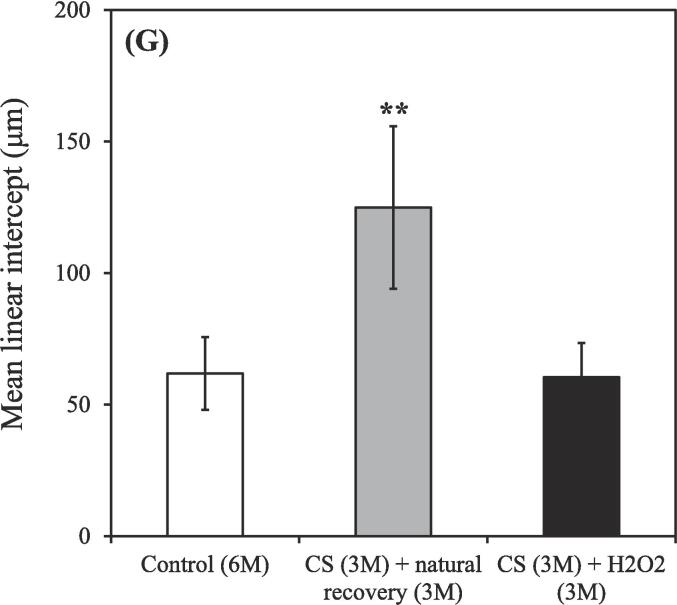

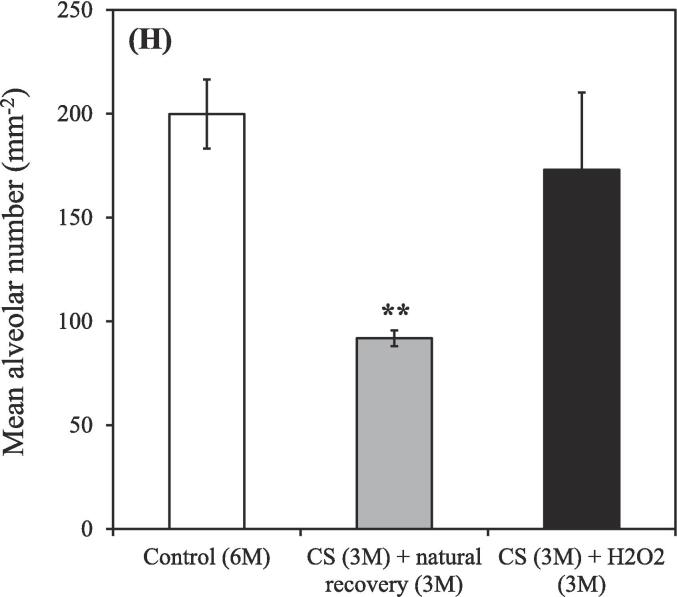
After 3-month CS exposure, the rats were subjected to different treatments and the histological changes were analyzed. (A) H&E staining of lung tissue obtained from the nonsmoking group (control) (red arrows indicate macrophages). (B) H&E staining of lung tissue obtained from the smoking group (CS) revealed an increased macrophage count, indicating an inflammatory response (red arrows indicate macrophages). Bars = 300 μm. (C) Lung porosity form both the smoking (CS) and nonsmoking (control) groups. [*p < 0.05 compared with the nonsmoking (control) group]. H&E staining of lung tissue obtained from both the smoking (CS) and nonsmoking (control) groups after the hydrogen–oxygen generator treatment. (D) Untreated nonsmoking group [Control (6 M)]. (E) Untreated smoking group [CS (3 M) + natural recovery (3 M)]. (F) Smoking group treated using the hydrogen–oxygen generator [CS (3 M) + hydrogen–oxygen (3 M)]. Bars = 200 μm. Morphometric measurements of pulmonary function were conducted to assess lung morphology. (G) Mean linear intercept: As alveolar spaces enlarged, the mean linear intercept increased. (H) Mean alveolar number: The number of alveoli in a given field of view. The groups were as follows: untreated nonsmoking group [Control (6 M)], untreated smoking group [CS (3 M) + natural recovery (3 M)], and smoking group treated using the hydrogen–oxygen generator [CS (3 M) + hydrogen–oxygen (3 M)]. (**p < 0.01, compared with the smoking group treated using the hydrogen–oxygen generator [CS (3 M) + hydrogen–oxygen (3 M)]). H&E, hematoxylin–eosin; CS, cigarette smoke.

The untreated nonsmoking group [Control (3 M)] exhibited relatively fewer macrophages (Fig. 4A), whereas an increasing trend in macrophage count was observed in the smoking group [CS (3 M)] (red arrows; Fig. 4B). Lung tissue affected by CS–induced COPD exhibited prominent inflammation (red arrows, representing circular, stained cells within alveoli; Fig. 4B). The lung porosity, as depicted in the re-plotted Fig. 4A-B, exhibited comparable outcomes [Control (3 M): 70.95 ± 5.38 %; CS (3 M): 79.11 ± 2.99 %] (Fig. 4C). We further observed the enlargement of damaged lung alveolar spaces, which were noticeably larger in the untreated smoking group [CS (3 M) + natural recovery (3 M)] than in the other groups (Fig. 4D–F), indicating the destruction of alveolar septa (Wang et al., 2014). However, treatment administered using the hydrogen–oxygen generator [CS (3 M) + hydrogen–oxygen (3 M)] ameliorated the damaged lung tissue (Fig. 4F), ensuring the recovery of alveolar spaces to a condition similar to that observed in the untreated nonsmoking group [Control (6 M)] (Fig. 4D).

The stained tissue slices (Fig. 4D–F) were quantitatively analyzed on the basis of the findings presented in Fig. 4G–H. The mean linear intercept (MLI) represents the average linear intercept of alveoli, with larger alveolar spaces corresponding to a greater MLI (i.e., smaller values are closer to normal). The mean alveolar number (MAN) represents the average density of alveoli, indicating the number of alveoli within a given observation field (a higher value indicates a more normal condition). After the 3-month CS exposure period + 3-month treatment period, the MLI values were as follows (Fig. 4G): 61.82 ± 13.82, 124.90 ± 30.91, and 60.41 ± 13.01 μm in the untreated nonsmoking group [Control (6 M)], untreated smoking group [CS (3 M) + natural recovery (3 M)], and smoking group treated using the hydrogen–oxygen generator [CS (3 M) + hydrogen–oxygen (3 M)], respectively. During this period, the MAN values were as follows (Fig. 4H): 199.83 ± 16.65, 91.87 ± 3.80, and 173.00 ± 37.19 mm^−2^ in the untreated nonsmoking group [Control (6 M)], untreated smoking group [CS (3 M) + natural recovery (3 M)], and smoking group treated using the hydrogen–oxygen generator [CS (3 M) + hydrogen–oxygen (3 M)], respectively.

### 3.5 Cardiac ultrasound results

The TAPSE value gradually decreased as the CS exposure time increased. Additionally, the smoking group treated using the hydrogen–oxygen generator [CS (3 M) + hydrogen–oxygen (3 M)] showed an increased TAPSE value compared to both the smoking group treated with a steroid [CS (3 M) + steroid (3 M)] and untreated smoking group [CS (3 M) + natural recovery (3 M)].

**Fig. 5:**
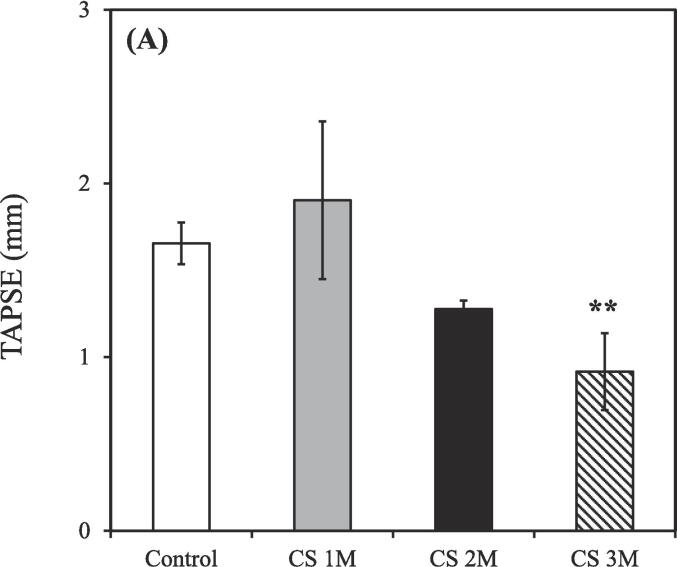

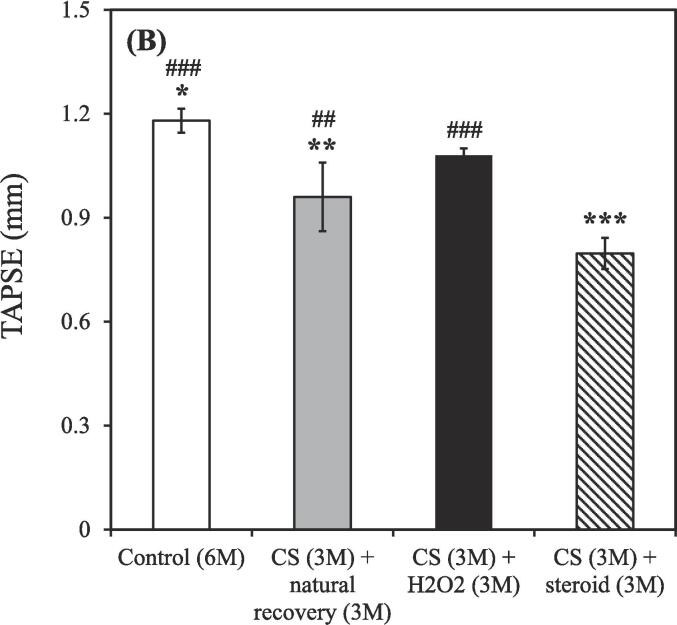
TAPSE values measured through ultrasound. (A) TAPSE values of rats exposed to CS for different durations. CS exposure duration: 1, 2, and 3 months. [**p < 0.01 compared with the nonsmoking (control) group]; (B) TAPSE values after treatment initiation. The groups were as follows: untreated nonsmoking group [Control (6 M)], untreated smoking group [CS (3 M) + natural recovery (3 M)], smoking group treated using the hydrogen–oxygen generator [CS (3 M) + hydrogen–oxygen (3 M)], and smoking group treated with a steroid [CS (3 M) + steroid (3 M)]. (*p < 0.05, **p < 0.01, ***p < 0.001 compared with the smoking group treated using the hydrogen–oxygen generator [CS (3 M) + hydrogen–oxygen (3 M)]; *^##^p* < 0.01, *^###^p* < 0.001 compared with the smoking group treated with a steroid [CS (3 M) + steroid (3 M)]). TAPSE, tricuspid annular plane systolic excursion; CS, cigarette smoke.

In the beginning of cardiac ultrasound, the TAPSE value in the nonsmoking group was 1.66 ± 0.08 mm (Fig. 5A). However, after CS exposure for 1, 2, and 3 months, the TAPSE values were 1.90 ± 0.45, 1.28 ± 0.05, and 0.92 ± 0.22 mm, respectively. Subsequently, treatment was administered. As shown in Fig. 5B, the TAPSE values were 1.18 ± 0.03, 0.96 ± 0.07, 1.08 ± 0.02, and 0.80 ± 0.05 mm in the untreated nonsmoking group [Control (6 M)], untreated smoking group [CS (3 M) + natural recovery (3 M)], smoking group treated using the hydrogen–oxygen generator [CS (3 M) + hydrogen–oxygen (3 M)], and smoking group treated with a steroid [CS (3 M) + steroid (3 M)], respectively.

### 3.6 Rationale for the difference in control groups

The control groups (0 M, 1 M, 2 M, and 3 M) were used as comparison groups to determine the effect of CS exposure on body condition (Fig. 2A-E, and 5A). Additionally, the control groups (0 M and 6 M) were used to assess the therapeutic effects of natural recovery, hydrogen–oxygen generator treatment, and steroid treatment, as well as to understand the impact of external environmental factors during the aging process (Fig. 3A-E, 4D-H, and 5B). Furthermore, the control group (3 M) was used to evaluate the success of the CS exposure model (Fig. 3F and 4A-C).

## 4 Discussion

Various agents, such as lipopolysaccharide, elastase, and CS exposure, can induce COPD in animals (Ghorani et al., 2017). Because the primary causes of COPD typically involve exposure to harmful particles or gases, particularly CS (Wei et al., 2024), establishing an animal model of CS–induced COPD is crucial for simulating real-life scenarios. Wang et al. indicated that as the CS exposure time increased, the pathological condition became more aggravated, peaking at 24 weeks (around 6-months) (Wang et al., 2018). However, the duration of CS exposure may result in increased mortality. Additionally, another study reported that only two months of CS exposure are sufficient to measure emphysematous changes that further progress on COPD rats (Leberl et al., 2013). Wang et al. also represented that rats exposed to room air for four weeks of recovery after eight weeks of sidestream smoke exposure showed therapeutic potential for COPD (Wang et al., 2018). This suggests that a natural recovery period of at least half the duration of the initial CS exposure (e.g., three months out of six) is necessary for therapeutic effects to manifest. On the other hand, Churg et al. demonstrated that after 3 months CS exposure, gene expression reached the minimum value for efficient repair, and by 6 months (when emphysema was present), gene expression levels were either at control levels or down-regulated below control levels. This indicates a failure to repair later in the disease (Churg & Wright, 2009). Thus, in the present study, we used three months of CS exposure followed by three months of recovery time.

Body weight was lower in the smoking group than in the nonsmoking group (Fig. 1A). This finding aligns with that of Wang et al. (2018). Interestingly, the food intake and water intake of rats in the CS group were higher than those in the control group within the first month (Fig. 1C and 1D). This may be due to emotional eating, which is a behavioral mechanism linking depression to the development of obesity and abdominal obesity (Konttinen et al., 2019). However, a previous study illustrated that upon inhalation, nicotine present in cigarettes rapidly enters the bloodstream and affects the central nervous system. This prompts the release of fatty acids from adipose tissue and increases the rate of fat oxidation, leading to an increased metabolic rate and elevated calorie expenditure, thereby affecting body weight (Audrain‐McGovern and Benowitz, 2011). These reasons could explain why the CS–exposed rats exhibited a higher appetite while their body weight still decreased. However, the aforementioned references describe human data, highlighting a limitation in that metabolic changes in rats following CS–exposure may not fully reflect those observed in humans. Further investigations will be conducted in future studies.

CS exposure for 3 months resulted in significantly increases in the counts of total white blood cells, neutrophils, lymphocytes, and monocytes, respectively (Fig. 2A-D), consistent with findings from Zheng et al. (2009), confirming the establishment of a CS–induced COPD rat model. However, a notable decrease in eosinophils in the BALF (Fig. 2E) was observed, likely due to the stronger association of eosinophils with asthma rather than COPD (Barnes, 2004, Matsumoto et al., 1998). Moreover, Liang et al. demonstrated that the count of white blood cells during the acute exacerbation of COPD was approximately 15 K/μL; however, during stable COPD, this count was approximately 8 K/μL (Liang et al., 2013). Considering that the total white blood cell counts for all groups were ≤ 10 K/μL (Fig. 2A), our COPD model falls within the range of the chronic phase.

To contextualize rat steroid exposures with human clinical corticosteroid dosing regimens, rat doses were converted to a HED using a body surface area conversion method. Based on this method, for a 60 kg adult, the corresponding human dose is approximately 12 mg (Equation (2). Although the value is slightly higher than previous findings, which show the utilization of inhaled corticosteroids in acute exacerbation of COPD at doses ranging from 2 mg to 8 mg daily (Gaude & Nadagouda, 2010), it is important to consider the significant differences in the patterns of CS inhalation between humans and rodents (Kabir et al., 2010, Mortola, 2004). Due to the highly developed and intricate nasal turbinates in rodents (Mess, 1999), they primarily inhale CS through their noses, whereas humans inhale smoke through their mouths. Furthermore, rodents exhibit avoidance behaviors and changes in breathing patterns when exposed to harmful substances. Thus, the above calculations suggest that the dosing conversion from rats to humans is plausible (administered dose for the rat: 1 mg/kg).

Although corticosteroids constitute a common therapeutic strategy for COPD, but they often lead to various side effects (McEvoy & Niewoehner, 2000). In contrast, oxygen therapy has gained traction as an alternative treatment for COPD (Mulhall & Criner, 2016). Recent findings from the pandemic period also suggest that hydrogen has antioxidative and anti-inflammatory properties, with the added benefit of being easy to administer (Li et al., 2023). Thus, evaluating the efficacy of hydrogen–oxygen therapy for CS-induced COPD is essential. As shown in Fig. 3, prominent decreasing trends were observed in the counts of total white blood cells, neutrophils, lymphocytes, and eosinophils counts in the BALF of the smoking group treated using the hydrogen–oxygen generator, confirming the therapeutic efficacy of the hydrogen–oxygen generator. Notably, an increasing trend, rather than a decreasing trend, was noted in the smoking group treated with a steroid. Although previous literature has demonstrated that dexamethasone reduces BALF lymphocytes, which contrasts with our recent results, those studies administered the drug intraperitoneally or orally (Sokar et al., 2021, Sadarani and Majumdar, 2015). In fact, steroids can be administered to humans via nebulization, intravenous injection, or oral routes (Zhang et al., 2018). Since our hydrogen–oxygen generator delivers the substance through inhalation for animals, we chose the nebulization method to maintain consistency with this mode of administration. Additionally, Barnes et al. demonstrated that inhaled corticosteroids offer limited clinical benefit in COPD due to the inflammation's resistance to corticosteroids (Barnes, 2010). While inhaled corticosteroids typically have minimal systemic side effects at standard doses, the higher doses used for COPD treatment can lead to systemic side effects, including an increased risk of pneumonia and exacerbation of systemic inflammation (Barnes, 2010, Marté et al., 2020). Furthermore, the total counts of white blood cells in the BALF increased dramatically after six months without any intervention in the untreated nonsmoking group [Control (6 M)] (Fig. 3A). This elevation is hypothesized to be influenced by external environmental factors during the aging process, as the body's immune response may trigger an increase in white blood cell count (Bauer et al., 2009). Similar trends were observed in neutrophil, lymphocyte, monocyte, and eosinophil counts, as shown in Fig. 3B-E.

An increasing trend was noted in the count of macrophages in the smoking group (Fig. 4B), indicating inflammatory reactions (Akata et al., 2020), confirming that smoking induces an inflammatory response, consistent with the findings depicted in Fig. 2. Additionally, Saetta et al. indicated that in smokers, alveolar walls are initially destroyed, followed by the enlargement of air spaces, thereby influencing lung function (Saetta et al., 1985). These findings are consistent with our results (Fig. 4C). Wang et al. conducted MLI and MAN measurements in animal models of COPD and found that although simvastatin can mitigate functional and structural impairment of the lungs, significant differences were observed in MLI and MAN between simvastatin-treated and control rats (Wang et al., 2014). In our study, the MLI values in the COPD rats decreased, while the MAN values increased after the hydrogen–oxygen generator treatment, compared with the values in natural recovery (Fig. 4G-H). Notably, no significant differences in MLI and MAN were observed between the untreated nonsmoking group [Control (6 M)] and the smoking group treated using the hydrogen–oxygen generator [CS (3 M) + hydrogen–oxygen (3 M)] (Fig. 4G-H). These findings suggest the superior benefits the hydrogen–oxygen generator against COPD, consistent with the staining results depicted in Fig. 4D–F.

TAPSE is associated with the lungs and is correlated with PaO_2_ values (Wagner, 2015). It quantifies the displacement of the tricuspid valve annulus during systole and serves as a tool for assessing the right ventricular function (Guazzi et al., 2013, Rosas et al., 2023). TAPSE helps detect potential damage to the right ventricular function in patients with COPD (Kharitonov et al., 2020). This is relevant because patients with COPD typically exhibit respiratory inflammation and lung tissue destruction, which disrupt gas exchange, leading to corpulmonale and compromising the right ventricular function (Karnati et al., 2021). Reductions in TAPSE values indicate impaired right ventricular function, suggesting a reduced ability of the heart to compensate for the pressure resulting from impaired lung function (Al Abbady et al., 2018). Prolonged CS exposure may induce pulmonary enlargement, manifesting as cardiac compression and increased pulmonary artery resistance, thereby reducing TAPSE values (Fig. 5A). Although the correlation between TAPSE values and COPD is yet to be clarified, patients with COPD tend to have relatively low TAPSE (Aloia et al., 2016, Vizza et al., 1998). However, after the hydrogen–oxygen generator treatment, the TAPSE values exhibited a trend toward recovery; the hydrogen–oxygen generator outperformed steroid treatment in conferring the recovery benefit (Fig. 5B).

Finally, we compared natural recovery (3 months of no therapeutic treatment) and therapeutic intervention—a hydrogen–oxygen generator—in terms of recovery after CS–induced COPD. Although the therapeutic efficacy of the hydrogen–oxygen generator is evident, natural recovery (smoking cessation) also exhibited a certain degree of therapeutic benefits against COPD–induced lung damage (Fig. 3A-B, 3D and 5B). Thus, smoking cessation remains an essential component of COPD treatment (Godtfredsen et al., 2008). Eliminating the pathogenic sources can mitigate the severity of COPD.

While our study has produced significant findings, it has limitations. Specifically, COPD remains underdiagnosed and misdiagnosed in humans. Data from the National Health and Nutrition Examination Surveys (NHANES) indicated that over 70 % of individuals with chronic airway obstruction lacked a formal COPD diagnosis (Ferrera et al., 2021). Furthermore, the potential effects of drug treatments in humans may vary depending on the stage of COPD (Churg & Wright, 2009). This complexity may partially explain the apparent failure of drug interventions in humans that appear to be effective in animals, as human interventions are typically administered late in the disease compared to early in the process in animal models. Whether the different phases (repair versus switching off of genes needed for repair) observed in animal models also occur in humans is not known (Churg & Wright, 2009), and the detailed mechanisms could be further elucidated in future work.

Finally, in cases where only two rats per group (n = 2) were used, the data should be considered preliminary and need to be replicated to ensure result reproducibility. Additionally, the results presented in this study are based on a single experiment, and further replication is required to confirm the efficacy of the hydrogen–oxygen generator for COPD in future research.

## 5 Conclusion

Our findings indicate that prolonged CS exposure causes lung damage in rats. This study provides experimental evidence for the association between smoking and COPD. We confirmed the efficacy of a hydrogen–oxygen generator in ameliorating COPD–induced lung damage in rats. This generator outperforms steroids in treatment of CS–induced COPD. The findings of this study may guide the development of future COPD treatments.

## Declaration of competing interest

The authors declare that they have no known competing financial interests or personal relationships that could have appeared to influence the work reported in this paper.

## Data Availability

Data will be made available on request.
