# Supplementary material for: Efficacy of a hydrogen–oxygen generator in treating cigarette smoke–induced chronic obstructive pulmonary disease in rats
Source: Curr Res Toxicol. 2024 Dec 28;8:100214. doi: 10.1016/j.crtox.2024.100214 (PMC11745982; doi:10.1016/j.crtox.2024.100214)
Supplement: Supplementary Data 1 [file mmc1.docx]

**Supplemental Figure Legends**

**Fig. 1.** Gas concentrations in the custom-made CS exposure device. (A) PM_2.5_ concentration; (B) Carbon monoxide concentration. In both the PM_2.5_ and CO graphs, noticeable downward peaks (indicated by red arrows) can be observed throughout the process. These fluctuations are due to the re-lighting of cigarettes during the experiment, which temporarily affects the concentration levels, causing a downward trend. However, the stability of our CS exposure device still proved beneficial for subsequent animal experiments.
